# Management practices to support donor transition: lessons from Avahan, the India AIDS Initiative

**DOI:** 10.1186/s12913-015-0894-0

**Published:** 2015-06-13

**Authors:** Sara Bennett, Daniela Rodriguez, Sachiko Ozawa, Kriti Singh, Meghan Bohren, Vibha Chhabra, Suneeta Singh

**Affiliations:** International Health Department, Johns Hopkins School of Public Health, 615 N Wolfe St, Baltimore, USA; Amaltas Consulting Pvt Ltd, C-20 Hauz Khas, New Delhi, 110016 India

## Abstract

**Background:**

During 2009-2012, Avahan, a large donor funded HIV/AIDS prevention program in India was transferred from donor support and operation to government. This transition of approximately 200 targeted interventions (TIs), occurred in three tranches in 2009, 2011 and 2012. This paper reports on the management practices pursued in support of a smooth transition of the program, and addresses the extent to which standard change management practices were employed, and were useful in supporting transition.

**Results:**

We conducted structured surveys of a sample of 80 TIs from the 2011 and 2012 rounds of transition. One survey was administered directly before transition and the second survey 12 month after transition. These surveys assessed readiness for transition and practices post-transition. We also conducted 15 case studies of transitioning TIs from all three rounds, and re-visited 4 of these 1-3 years later.

**Results:**

Considerable evolution in the nature of relationships between key actors was observed between transition rounds, moving from considerable mistrust and lack of collaboration in 2009 toward a shared vision of transition and mutually respectful relationships between Avahan and government in later transition rounds. Management practices also evolved with the gradual development of clear implementation plans, establishment of the post of “transition manager” at state and national levels, identified budgets to support transition, and a common minimum programme for transition. Staff engagement was important, and was carried out relatively effectively in later rounds. While the change management literature suggests short-term wins are important, this did not appear to be the case for Avahan, instead a difficult first round of transition seemed to signal the seriousness of intentions regarding transition.

**Conclusions:**

In the Avahan case a number of management practices supported a smooth transition these included: an extended and sequenced time frame for transition; co-ownership and planning of transition by both donor and government; detailed transition planning and close attention to program alignment, capacity development and communication; engagement of staff in the transition process; engagement of multiple stakeholders post transition to promote program accountability and provide financial support; signaling by actors in charge of transition that they were committed to specified time frames.

## Background

If the health services provided by donor-funded programs are to be sustained, then at some point in their lives such projects will need to transition to local ownership, meaning that responsibilities for both funding and running the program will be taken over by local actors. The stimuli for such transitions vary: perhaps economic growth means that the country no longer needs donor support, or donor priorities have shifted to other issues. Regardless of the reasons for transition, the transition process itself may be challenging with a risk of disruptions to services, and in the most extreme instances, their discontinuation. This paper aims to identify management practices that support effective transition, and explore why particular practices appear to be successful.

The concepts of transition, country ownership and sustainability can be confusing, and are not explained in a consistent fashion in the literature. Often the overarching goal is to ensure program sustainability, whether this is measured as maintenance of health benefits or the continued supply of services [1, 2]. While the term country ownership has been very diversely defined in the development literature [3], we understand it as the ability of actors (both public and private) within the country to provide vision and leadership, financial support and implementation capacity to manage a program. There may be varying degrees of country ownership [4], for example a country may provide strong political leadership to a program but not be able to provide sufficient financing for that program. Transition is the process through which greater country ownership and sustainability are achieved. There are different ways in which a transition can be thought to be ‘”effective”. For example a transition may be particularly effective at promoting country ownership, or ensuring the sustainability of health outcomes. In this paper our focus is on the short term. We examine how management practices support a smooth transition, that is, services are handed over to local owners with minimal disruption. While a smooth transition is not the ultimate goal of transition, it appears likely to be on the critical pathway to sustainability, in the sense that services may never recover from disruptive transition processes.

Several papers have sought to describe what should be done to support an orderly program transition that maximizes the likelihood that programs will be sustained post-transition. Factors typically highlighted include the need for high level political leadership of transition; close communication and coordination with local partners, particularly government; early planning for transition; strategies to build local organizational capacity; and a phased approach to transition [5–8]. However, most such papers focus primarily on negotiation of the overall transition process with local stakeholders (primarily government) rather than operational aspects of transition management. These questions are becoming particularly pressing as the US Presidential Emergency Plan for AIDS Relief (PEPFAR) increasingly seeks to transition its HIV programming [9]. Early evidence from South Africa suggested that there were multiple disruptions to antiretroviral programs as PEPFAR transitioned, including stock-outs of ARV and TB drugs, long wait times, and poorly prepared staff [10]. Agency reports are seeking to provide guidance on good management practices during transition processes [11, 12], but typically these are based on anecdotal cases or prospective analysis, rather than rigorous research, as there is an extremely limited research base to support understanding of this question.

In thinking about management practices which might support program transition we drew upon the change management literature, particularly that which reflected the operational challenges relevant to health programs [13]. Change management, like transition management, concerns high-level organizational change that embodies the organization’s strategic goals. Further, change management focuses in particular on the human and relational aspects of change, which prior reports in this field have suggested to be important [14]. While there is substantial debate within the change management literature about the relative merits of intermittent versus continuous change, and planned versus emergent change, there appears to be greater consensus regarding practical guidance for emergent change. Both studies specific to the health sector [15, 16] as well as broader change management frameworks [17, 18] emphasize a number of key themes including:-

i. ***Developing a vision, and mobilizing commitment and leadership for change*** – create a clear vision and common direction, and develop effective individual or distributed leadership for change
ii. ***Crafting an implementation plan or strategy*** –develop supportive organizational systems and structures
iii. ***Engaging staff*** – identify the human consequences of change and take actions to address these consequences; align staff with organizational goals; develop necessary skills for them to operate effectively in the new organization
iv. ***Communicating and informing stakeholders*** – encourage stakeholder participation and empower broad-based action; bridge intra- and inter-organizational divides so that everyone knows what is going on and can work together
v. ***Generating short term wins*** – maintain a focus on results; monitor results and adjust strategy if problems appear to arise; disseminate early wins so as to ease the way for further change.

### Transition of the Avahan HIV/AIDS prevention program

Our data come from an assessment of the transition of the Avahan HIV/AIDS prevention program in India [19]. Between 2003 and 2012 the Avahan Initiative, supported by the Bill and Melinda Gates Foundation (BMGF), worked in Andhra Pradesh, Karnataka, Maharashtra, and Tamil Nadu (as well as two states in north-east India) to provide HIV/AIDS prevention services to high risk groups (HRG). In each state, a State Lead Partner (SLP), either an international Non-Government Organization (NGO) or a large domestic NGO, oversaw and supported a series of Targeted Interventions (TIs) that were run by local NGOs that provided services to key populations (primarily female sex workers, men who have sex with men, and transgenders) including peer education, testing and treatment for sexually transmitted infections (STIs) (often accompanied by other general health services), distribution of condoms, sensitization of health care providers and police, and community mobilization [20, 21]. Individual TIs (numbering approximately 200 in total) were transferred to government under the oversight of State AIDS Control Societies (SACS) in their respective states, with 10 % of the TIs transitioned in 2009, approximately 20 % in 2011 and the remaining 70 % in 2012 [22]. Table 1 provides an overview of key Avahan and government actors in the transition process at multiple levels of the health system.

**Table 1:** Key actors in the Avahan initiative transition process

Level	Government	Avahan*
National	National AIDS Control Organization (NACO) responsible for strategy and oversight of national AIDS control program	Bill and Melinda Gates Foundation (BMGF) staff directly involved in the design and management of the Avahan Initiative
State	State AIDS Control Societies (SACS) government actors that oversee HIV/AIDS services within the state and manage contracts with organizations providing targeted interventions Technical Support Units (TSU) contracted units designed to provide technical support to HIV prevention programs	State Lead Partners (SLP) international NGOs or large domestic NGOs that manage contracts with and support targeted interventions under Avahan Bill and Melinda Gates Foundation (BMGF) – one designated staff providing oversight of each state program.
District and Community level	District AIDS Prevention and Control Units (DAPCU) responsible for district level planning and monitoring Targeted interventions - NGOs or Community Based Organizations contracted by SACS to provide HIV/AIDS prevention services	Targeted interventions – NGOs or Community Based Organizations contracted by State Lead Partners to provide HIV/AIDS prevention services

*We use the term “Avahan” to refer to the whole array of partners involved in implementing the Avahan initiative

From early on, BMGF staff recognized that the program should ultimately be transitioned to its local owners. This was reflected in a preliminary 2006 and more detailed 2009 Memorandum of Cooperation between Avahan and the National AIDS Control Organization (NACO) that clearly stated the time frame and tranches for transition [23, 24]. Further, the National AIDS Control Programme III (NACP III) [25] provided a policy framework that reflected the activities that Avahan TIs had been undertaking, and indeed the government was already supporting many of its own TIs to provide the same services. Finally, lack of funding was not a problem, with a total budget for NACP III activities of US$2.5 billion, approximately two thirds of which was allocated to prevention, with one third of this targeted at high risk groups, the program was relatively well funded by government [26]. In light of these factors, it is safe to say that there was strong national policy level support for the transition process – but by 2009 the greatest challenge lay in operationalizing the transition at the state and local level. Our analysis of management practices focuses primarily on the TI level and the state level actors who supervised and supported TIs.

## Methods

We conducted a prospective evaluation of the transition of the Avahan program during the period 2009–2013. This paper describes management practices during the three rounds of transition in 2009, 2011 and 2012. While the first transition tranche (2009) was implemented quickly, with limited planning, there was considerable time for transition preparation and careful management of transition in the second and third rounds. This contrast between the first relatively poorly managed round, and the better managed second and third rounds allows us to analyze the management practices adopted over time, and how they contributed to a smoother transition.

In presenting our analysis of management practices we draw upon three study components:

- *Transition readiness survey* - a quantitative assessment of the “transition readiness” of all 27 TIs that transitioned in 2011 and a sample of 53/154 TIs that transitioned in 2012, randomly selected at the state level with number selected proportional to size. This assessment was conducted through a structured survey with some open-ended questions administered to the managers of the TIs. The survey examined critical areas of transition readiness, encompassing communication of the transition process, capacity building for transition and alignment of the program with government guidelines, using primarily objective questions that explored for example, the number of staff who had been trained for transition, which aspects of services had been aligned, and to whom news about the transition had been conveyed. Where possible, responses were checked against objective sources (e.g. Annual reports)
- *Follow-up survey* - the same sample of TIs that had been surveyed for transition readiness were surveyed again approximately 12–18 months after transition had occurred, using a structured survey with some open-ended questions. Like the transition readiness survey this was administered to the managers of TIs, and included a mix of opinion-based questions (e.g. Are government actors as committed to HIV prevention services as Avahan was?) and fact-based questions that sought to examine the maintenance of various management practices after transition. Again, where possible, responses were checked against documentary data. A smaller number of TIs were surveyed in the follow-up survey (N = 70) as some TIs had been merged with others.
- *Longitudinal case studies* - case studies of 15 TIs were conducted shortly after the transition process was complete. Four of these TIs were revisited for further data collection one to three years later. The case studies were selected so as to maximize variation across states and the type of key population served. Primarily qualitative data were collected, and within each TI in-depth interviews were conducted with TI managers, representatives of the Avahan SLPs (who had managed the program under Avahan) and SACS (who managed the program once government took over), as well as focus group discussions with key populations, including peer outreach workers. In total, across the 15 TIs, we conducted 83 in-depth interviews and 45 focus group discussions. These interview data were supplemented by relevant TI documents and routine health information data.

Table 2 provides a summary of the numbers of TIs sampled for each of the study components, by transition round. All instruments were administered by trained Indian researchers who used appropriate local languages.

**Table 2:** Sample of Targeted Interventions (TIs) for structured surveys and case studies

	Andhra Pradesh	Karnataka	Maharashtra	Tamil Nadu	Total
Transition preparedness survey (before transition)					
2011 Round	11	6	6	4	27
2012 Round	16	17	13	7	53
Follow-up survey (12 months after transition)					
2011 Round	11	5	8	4	28
2012 Round	13	12	13	4	42
Case studies					
2009 Round	1	1	1	1	4
2011 Round	1	2	1	1	5
2012 Round	2	1	2	1	6
Revisits	1 (2009)	1 (2009)	1 (2011)	1 (2011)	4

Quantitative data and short open-ended responses to the transition readiness and follow-up surveys were translated into English, and entered into and analyzed in Excel. Analysis sought to compare the degree of transition readiness, and perspectives on the outcomes of transition by round, by state and by population served.

Qualitative data from the case studies were transcribed, translated and analyzed using Atlas.ti. Themes in the data were initially coded according to key issues in the overarching research protocol that included transition preparedness, changes to services, the smoothness of transition, lessons learned, and relationships between different actors. Sub-codes within these themes focused on specific issues, such as staff training for transition, changes in budget, staffing etc. A case study profile of each TI was constructed combining qualitative and quantitative data. This paper draws findings from across the 15 case studies, identifying key findings around the aspects of the change management framework identified below.

This study was ethically reviewed by Johns Hopkins School of Public Health Institutional Review Board and exempted. Within India it was reviewed and approved by YRG Care Institutional Review Board.

## Results

Findings are organized around the five practical themes for change management identified above.

### Develop a vision, and mobilize commitment and leadership for change

The vision behind the Avahan transition was set at the national level by BMGF and NACO as articulated in the 2006 and 2009 Memoranda of Cooperation [23, 24], but effective implementation of that vision required the mobilization of commitment and leadership for change throughout the system. In the first round of transition, 2009, it was clear that there was not a sense of joint leadership or a vision for change that was shared by Avahan actors, and state-level government actors. Instead, the 2009 transition process was frequently characterized by animosity and mistrust between Avahan and government actors. For example, one respondent in government described how he had heard TI staff referring to the transition as an arranged marriage:

*“A love marriage would be about preference or liking. In an arranged marriage you have the partner - may be it is forcefully, so you have to like the partner whom you have married. So this kind of comment was not very well taken”* Maharashtra, Government, 2009 Round.

In the first round this sense of mutual mistrust and lack of buy-in to the transition process manifested particularly strongly with respect to baseline evaluations undertaken by NACO prior to the transition in order to assess the quality of TIs it was taking over.

*“NACO said that whichever TI we are going to take, we will evaluate them first and this evaluation would be done centrally to understand what Avahan has been doing…… So, that exercise was done by NACO in consideration with the SLPs. In Karnataka also the same thing happened and the TIs were evaluated and some of these evaluation reports were also pretty depressing. It did not paint a very good picture. And there were also issues between the SACS people and [name of NGO] as well. So, there were other interpersonal issues which also came up in these reports.”* Karnataka, Government, 2009 Round,

However, in the 2011 and 2012 rounds of transition, both SACS and Avahan worked towards much more trusting and mutually respectful relationships, with leadership of the transition process effectively shared between both partners.

*“Transition doesn’t happen only because of a few people working for it, what I feel is that transition primarily happens if you have a good system and a good rapport in place….So the most important thing that I realized in this doing the transition process is that good personal touch or interpersonal relations matter a lot during transition, doing such a big thing like the transition… I feel that if you don’t have good interpersonal relations at a higher up level then like anything can go wrong at any point of time”.* Maharashtra, SLP, 2011 Round

*“They [SACS] have cordial relationship with us. Most of the time we will resolve the issues…., jointly we go and address…So any issues immediately they call me, or I do call them. So immediately we will address the issues.”* Tamil Nadu, SLP, 2012 Round

Although the 2011 and 2012 rounds of transition were not free of troubles or tension, particularly when staffing transitions meant that new relationships had to be forged, the foundation of strong relationships between key actors supported joint problem-solving. Through the progressive rounds of transition, enhanced commitment, clear joint leadership and greater role clarity developed among state level actors in most cases. In turn, this appeared to contribute to a growing sense among TI level staff that SACS provided clear leadership to the HIV/AIDS prevention agenda (see Table 3).

**Table 3:** TI manager perceptions of government commitment toward the program 12 months after transition (follow-up survey)

		Strongly Agree	Agree	Neutral	Disagree	Strongly disagree	No Response
SACS has the same or a higher level of commitment toward the program as compared to Avahan	2011 Round	18 %	64 %	11 %	4 %	0 %	4 %
	2012 Round	29 %	48 %	14 %	7 %	0 %	2 %
The NGO/CBO and SACS share a common vision for HIV prevention	2011 Round	86 %	14 %	0 %	0 %	0 %	0 %
	2012 Round	83 %	14 %	0 %	0 %	0 %	2 %

### Craft an implementation plan or strategy

In the 2009 round of transition there had been very limited scope for implementation planning given the tight timelines. Often debates about which TIs should transition in the first round were still ongoing immediately preceding transition. In some instances it appeared that there was also a lack of appreciation for the level of effort, time and planning that transition would entail, from all actors:

*“See, at that point of time, there was very little experience on transitioning itself, at that point of time, there was pretty much no national guideline on formats, you know and things like that. … the thing is that we all felt that the programme could just be taken over. The only thing which we would need to do would be to actually re-align the clinic programme. So, rest of the things are already functioning within the TI settings. So, we did not really see much of a challenge and not much of, what do you call, transitioning preparedness as compared to now.” Karnataka, Government, 2009 Round*

This lack of planning during the first round manifested itself in several ways. There were no budget lines in the Avahan budget dedicated to transition, nor staff with a particular mandate to focus on transition. Communication between concerned stakeholders was often poor (as described above). Government contracts typically did not materialize until several months after the formal transition handover and during these months TIs struggled with financial flows, and procuring condoms and other supplies. While there was recognition of the need to align clinical services, management and reporting structures with government norms, frequently this process was incomplete at the time of transition. Even if salaries and budgets had been aligned, TIs had not had sufficient time to adjust to the new funding processes.

Also, during the first round of transition, arrangements for SLP support to TIs after transition were contentious. There were no formal agreements in place for post-transition support, and in some states SACS asked SLPs to cease contact with TIs after transition. However, during the second transition round, this issue was tackled directly and post-transition support agreements between the SLP and SACS were negotiated prior to transition. These agreements clearly specified what support the SLPs would provide to TIs after transition and this helped clear up confusions around roles.

Indeed in the run up to the second (2011) round of transition, Avahan developed a much more structured and planned approach. Each SLP hired a transition manager responsible for overseeing the transition process, and a national level transition manager was also appointed by BMGF. Transition plans were developed with dedicated budgets to support transition processes. Based upon the transition readiness surveys, preparation for transition started much earlier: during the 2011 round, efforts to prepare TIs for transition started on average 6 months before transition, and for the 2012 round this increased to 9 months. Transition preparation efforts encompassed staff training to prepare people for the transition, alignment of both clinical and administrative structures, as well as the accumulation of buffer stocks in case of a gap in supplies and financial reserves at the time of transition. By the third transition round (2012) these elements had been formalized by Avahan in a “common minimum programme” [27] for transition that was used to guide the transition preparation process across the large number of transitioning TIs (Table 4).

**Table 4:** Core elements of Avahan common minimum programme for transition

Nature of Activity	Description	Responsible Actors	Timing
Planning	Develop a step-by-step plan for each round of transition with clear milestones. Agree on this with SACS.	SLP, transition manager, SACS, TSU^1^	12 months prior to handover
Selection of TIs	Develop criteria to select TIs to be transitioned.	SLP, SACS, TSU	12 months prior
	Review core program indicators of potentially transitioning TIs	M&E Manager, transition manager	6 months prior
	Conduct mock joint assessments	SLP	3-6 months prior
	Conduct joint assessment visits to finalize selection	SLP, SACS, TSU	3-6 months prior
Staff orientation	Training for SLP staff, CBO and NGO staff and district officers in different aspects of transition	SLPs	6, 3 and 1 month prior
Alignment	Align Avahan TI contracts with government norms	SLP	Started earlier over rounds: approx. 12 months prior in 2012 round
	Ensure TIs follow all government administrative norms (Budget, staffing etc.)	SLP	1 year prior
	Ensure TIs following government reporting system	SLP	6-12 months prior
Develop post-transition support agreements	Develop a clear post-transition support document, with clear levels of budget and deliverables	SLP and SACS	1-2 months prior
Back-up support	Create a financial reserve in case of delays in funding disbursement	SLP	As per need at handover
	Deliver support as agreed in post-transition support document	SLP	As agreed in post-transition support document
Ongoing monitoring	Monthly joint visits with TSU and SLP to provide monitoring and mentoring support	SLP	As agreed in post-transition support document

Acronyms: CBO – Community Based Organization; NGO – Non-Government Organization; SACS – State AIDS Control Society; SLP – State Lead Partner; TI –Targeted Intervention; TSU – Technical Support Unit

^1^TSU – Technical Support Unit

Source: Avahan Common Minimum Programme for Transition

Significant effort was invested in aligning both clinical and non-clinical elements of the program. For example, most clinical services had to be shifted from direct delivery by the TIs (e.g. through clinics at the drop-in-center) to referrals to government health facilities. Frequently SACS sought to sensitize workers at government clinics prior to or at the time of transition so that they behaved in a manner that did not stigmatize or discriminate against the HRGs. Further, TIs had to switch their procurement channels so that they procured medicines for STIs and condoms from government suppliers rather than the SLP. Overall, by the second round, these shifts in the clinical and non-clinical management of the program appeared to be handled successfully. The transition readiness study showed relatively high alignment of Avahan TIs with government norms at the time of transition for both the 2011 and 2012 rounds (Table 5). Where alignment was not high e.g. for the procurement of STI syndromic management medicines, this was due to the fact that TIs had built up buffer stocks, thus delaying the shift to procurement through government systems. A similarly positive picture emerged with respect to alignment of non-clinical services, including budgets, TI team structures and reporting systems.

**Table 5:** Alignment of Avahan TIs with government clinical and non-clinical norms at the time of transition

	Round 2 (2011)			Round 3 (2012)		
	Low	Medium	High	Low	Medium	High
Is the NGO/CBO following the STI syndromic management guideline of NACO?^1^	15 %	0 %	85 %	2 %	13 %	85 %
Does the NGO/CBO procure STI syndromic management medicines as per NACO/SACS guidelines?^2^	37 %	7 %	56 %	53 %	9 %	38 %
Has there been any change in the condom procurement process?^3^	26 %	7 %	67 %	15 %	11 %	74 %
Has there been any change in the budget as per NACO/SACS guidelines?^4^	0 %	7 %	93 %	2 %	11 %	87 %
Has there been any change in the reporting format: are you sending reports to SACS/District AIDS Control Societies?^5^	0 %	4 %	96 %	8 %	2 %	89 %
Has there been any change in team structure: are you following SACS/NACO guidelines?^6^	0 %	7 %	93 %	0 %	4 %	96 %
How aligned is the present ratio of peer educators to high risk groups with the SACS norms?^7^	0 %	4 %	96 %	0 %	8 %	92 %

^1^Low: Avahan guidelines are still in place; Medium: Some changes are made according to NACO guidelines; High: Following STI syndromic management guidelines of NACO.

^2^Low: Avahan supply chain is still in place; Medium: Some changes are made in the procurement processes as suggested by NACO/SACS; High: STI syndromic management medicines are procured as per NACO/SACS guidelines

^3^Low: Process not initiated; Medium: Some changes are made in the condom procurement processes; High: All condom procurement is done through channels suggested by SACS

^4^Low: No change, still following Avahan budget; Medium: Some changes were made to the budget; High: Following NACO/SACS budget guidelines

^5^Low: No change in reporting format; Medium: SACS formats discussed but not all introduced; High: Following all SACS formats

^6^Low: No change in team structure; Medium: Some changes were introduced; High: Following SACS TI structure

^7^Low: Ratio was not previously measured; Medium: Ratio is measured and approaching that of SACS; High: Following the SACS ratio

### Engage staff

Communication among upper and middle-level managers was typically very good in the 2011 and 2012 transition rounds, but front-line staff tended to be less well informed about transition. As the Avahan transition Common Minimum Programme suggests, in the 2011 and 2012 rounds a considerable amount of effort was targeted at training TI staff for their roles post-transition and communicating the transition process to staff members. Findings from the transition readiness survey suggest the numbers of meetings held prior to transition increased between 2011 and 2012, with greater involvement by lower level staff.

There were improvements over time in the amount of training on transition provided to TI staff. For example in 2012, approximately 70 % of transitioning TIs had two or more staff members trained in the different transition modules (TI guidelines, programming, outreach planning, condom programming, community mobilization, communication etc.) compared to 55 % of TIs in the 2011 transition round and virtually none in Round 1. It remained the case, however, that higher level staff were more likely to receive training than lower level staff, and for the 2012 round, some staff training took place as late as March, just one month before transition.

While strong efforts were made to develop skills and align the individual goals of staff with the goals being pursued through transition, it was difficult to address the broader consequences of transition for staff, particularly the fact that government budgetary norms typically paid lower salaries for TI staff than under Avahan. Frequently the shift in salary structures was made in advance of transition as part of the alignment process. This led to reductions in salary levels although the magnitude of the drop in salaries varied across states and across TIs. There was just one exception to this, in the 2011 round of transition in Tamil Nadu the SLP managed to negotiate with SACS for higher salaries for TI staff, based on their extensive experience. While the drop in salaries led to some staff dissatisfaction and staff turnover, in general the consequences appeared to be less than many SLPs or SACS feared.

*“It was totally aligned with SACS salaries in March 2010 [two years prior to transition]. We had some staff turnover when we aligned but it was less than what we anticipated. We were very pleasantly surprised. We had this notion, let me be very honest with you, before aligning we had this notion that “oh! what will happen? Salaries are so low what will happen. Will people leave? If there is a mass staff attrition, what we will do?” We had all those concerns. In fact, this was one of the most important concerns we had. But when we actually sat down in this room to restructure the TIs and align them, we found that there were these concerns but there were opportunities as well in the realignment process.”* Maharashtra, SLP, 2012 Round

Staff drawn from focal communities (eg. peer educators) were also affected by lower salaries (as discussed in Rodriguez et al. forthcoming [28]).

### Communicate and inform stakeholders

Managing the mechanics of program transition took a very high level of investment from Avahan and government staff, involving repeated meetings between SLPs and government counterparts in NACO, SACS, and Technical Support Units at the state level, and District AIDS Prevention and Control Units. Avahan appeared to prioritize this effort with government over engagement with other stakeholders.

However, Avahan, and in particular the BMGF staff, perhaps missed opportunities to broaden ownership of the program by focusing primarily on government stakeholders. In articulating the goal of transition, the original transition plan [29] noted three different elements: transition to government; development of a coalition of partners to sustain impact; and community action to sustain impact. There were multiple stakeholders in the Avahan program beyond government HIV/AIDS organizations, including HRG community based organizations, other government departments that interacted with the TIs such as police departments, and a broader array of civil society organizations concerned about the rights of HRGs and their access to services. In practice, Avahan efforts to transition focused primarily on the government HIV/AIDS organizations and paid little heed to other stakeholders, although in some cases the mere fact of integrating Avahan TIs into the government system appears to have strengthened ties with other government departments.

While Avahan recognized the importance of mobilizing communities to sustain impact, community mobilization proved the most difficult aspect to transfer to government particularly given limited or non-existent government budgets for such activities. Our follow-up surveys showed that one year after transition only 64 % of the community groups supported by the TIs that transitioned in 2011 and 45 % of those supported by TIs that transitioned in 2012 had found additional sources of income. For the community groups associated with the 2011 TIs, these revenues were entirely internal (membership fees and income-generating activities), although for the 2012 round some community groups received support from local foundations. In 2012 Avahan developed a third and final phase, with the aim of providing ongoing community mobilization support to the community groups associated with the transitioned TIs.

### Generate and disseminate short term wins

While there were many good reasons to phase the transition process, the 2009 round of transition though small had been very challenging, thus leading to few obvious “short term wins” or success stories that could be disseminated. Conversely, the second round had been much more successful, but there was limited time between the completion of the 2011 and 2012 rounds to document and disseminate success.

The lack of early “wins” does not appear to have been a significant barrier to the overall program of transition. Respondents from TIs rarely cited other Avahan transition experiences in their state, whether positive or negative, and there appeared to be little direct communication between TIs. However SLPs and BMGF program officers clearly interacted and shared information about transition and sometimes multiple TIs were managed by a single NGO, which also enabled learning across TIs. Overall, problems during the first transition round appear to have led to greater efforts between state-level actors to communicate effectively, suggesting that it sparked positive learning and action cycles [30].

While there were not obvious short term wins from the first round of transition, its failures sent a clear message to both SLPs and NGOs running TIs that transition was certain and would proceed, even if conditions were not ideal. This may have been an important message in terms of reinforcing commitment to transition, given that for both these groups of stakeholders, maintaining the status quo was probably easier than transitioning.

## Discussion

Building on change management frameworks, this study sought to document and assess the main management practices associated with the transition of the Avahan program from BMGF to the government of India. The transition process was completed in a timely fashion, and our studies of transition readiness indicated relatively high levels of program alignment, effective communication and capacity building in both the 2011 and 2012 transition rounds. Further, we found that 9 months after transition, key indicators of program performance were sustained. Therefore, we believe that the management practices described here facilitated a relatively smooth and successful transition process.

After experiencing a multitude of problems associated with the 2009 transition round, Avahan invested major efforts in developing and implementing operational plans for transition. Key elements of these transition plans included the establishment of transition managers in each SLP and budget lines to support their activities, negotiation of detailed state level transition plans, regular joint supervisory visits (between Avahan and government) to help TIs align with government standards, build-up of buffer stocks and establishment of post-transition support agreements. Communicating the goals of transition to staff and training them in operational practices post-transition were also important elements of transition management plans.

However, the detailed management plans described above would not have been developed or implemented, if it were not for the establishment of trusting and transparent relationships between government and Avahan state-level actors. While some of the good management practices such as staff training could have been implemented by Avahan alone, most needed to be negotiated, agreed and jointly implemented by Avahan and government. The main differences between states in their management of the transition arose from differences in the quality of relationships between government and Avahan actors at the state level. While in three of the states the relationships became progressively stronger, there was one which suffered setbacks due to staff rotations.

While this paper has sought to identify transition management practices that cut across different TIs, in practice there were many nuances that emerged according to the nature of state level relationships (as described in the paragraph above) as well as features of individual TIs. For example, there were differences between those TIs that transitioned as NGOs, and those that transitioned as Community Based Organizations (CBOs). NGOs were typically better prepared for transition, but CBOs tended to be better at informing and engaging their staff. These differences highlight the critical importance of local context to managing effective transition, and suggest that management strategies will likely need to be adapted to reflect different conditions on the ground.

The lengthy timeline and the phased nature of the transition stand out as being critical enablers of the good management practices identified. Through planning transition in three increasingly large tranches it was possible to gradually strengthen relationships as well as facilitate learning. Respondents recognized the importance of learning from previous rounds, but also the unpredictability of the process, as one commented: *“you plan for ten things, transition throws eleventh thing to you”*(Karnataka, SLP, 2011 Round). One caveat to the phased transition design was that some respondents thought that the final transition round, with 70 % of TIs, was too large, and not all of the management strategies developed in previous rounds could be effectively applied with such a large group. These respondents suggested that the size of the second round could have been increased so as to make the final round more feasible to transition effectively.

While transition management appeared broadly successful, one area that was perhaps relatively overlooked by Avahan, particularly BMGF staff, was communication with a wider array of stakeholders. Avahan’s intensive engagement with government appeared to limit the appetite for engaging other potential partners such as development partners, funders, or civil society organizations. This inclination to focus on core business, at the expense of building a broader coalition for transition, primarily reflects extensive demands on staff time to manage the transition process, but may have been reinforced by the departure of some senior BMGF staff prior to the full transition of the program. While BMGF has sought to strengthen community engagement during the past two years, a broader ownership of the transitioned TIs might help bolster sustainability.

Finally, our analysis suggests that the need to secure and disseminate “early wins” was not as important to the Avahan transition process as change management frameworks suggest. A number of reasons may explain this. First, this study does not address change within a single organization where news may travel fast about successes and failures, but rather across a broad, geographically distributed, health system. Second, the ragged 2009 transition round perhaps served to focus people’s minds and convince them of the need for a more structured and planned approach, which may have been an unexpected benefit. Third, and perhaps most importantly, the fact that the 2009 transition round went ahead despite inadequate preparation may have been critical in convincing partners that BMGF was committed to the identified timelines and transition would not be derailed by lack of preparation.

### Limitations

This paper has not linked the discussion of management practices to evidence on the long-term sustainability of the Avahan program but has instead focused on how smoothly transition progressed. We focused primarily on state and TI level actors rather than the national level, reflecting that for Avahan, many policy decisions regarding transition were resolved by 2009, in other contexts achieving high-level agreement around transition may be much harder.

A number of additional factors may limit the transferability of the Avahan transition management lessons. First, Avahan addressed prevention needs of HRGs in a concentrated epidemic, rather than for a generalized epidemic: a generalized epidemic would warrant a larger scale of activities, but government may find it easier to meet the general population’s needs compared to HRGs’ needs. Second, there has been substantial growth in the Indian government’s budget for HIV/AIDS since 2004, which means that there were adequate financial resources to support transitioned programs. Third, the NACP III provided a policy framework for HIV/AIDS prevention among HRGs in India, which closely matched the principles of the Avahan program [31]. Finally, there are relatively high levels of bureaucratic capacity across the Indian government’s HIV/AIDS administration. Despite these somewhat distinctive features of the Avahan transition, we believe that many of the management lessons are transferable.

## Conclusions

Our findings suggest that management of donor transitions is likely to be stronger when:

- There is an extended and sequenced time frame for transition that facilitates both learning and development of trusting and transparent relationships
- Transition processes are co-owned and planned by both the donor and the government
- Detailed transition planning takes place, supported by clear guidelines, and close attention is paid to program alignment (both financial and operational) and capacity development
- Transition processes are supported by dedicated staff and transition budgets
- Staff are engaged in the transition process, and understand both the goals of transition and the likely consequences of transition
- Clear communication about the new arrangements is provided including participation by staff in decisions about changes in the structure of support.
- A broad array of stakeholders are engaged to support the program post transition through enhanced program accountability and additional financial support
- Actors in charge of transition signal that they are serious about it and will not be derailed by poor preparation.

## Abbreviations

AIDS: Acquired Immunodeficiency Syndrome
BMGF: Bill and Melinda Gates Foundation
CBO: Community Based Organization
HIV: Human Immunodeficiency Virus
HRG: High Risk Group
NACO: National AIDS Control Organization
NACP: National AIDS Control Plan
NGO: Non-government organization
PEPFAR: Presidential Emergency Plan for AIDS Relief
SACS: State AIDS Control Society
SLP: State Lead Partner
STI: Sexually Transmitted Infection
TI: Targeted Intervention
TSU: Technical Support Units
